# Methodological considerations in a nutritional intervention trial among patients receiving methadone maintenance treatment

**DOI:** 10.1016/j.dadr.2026.100489

**Published:** 2026-10-30

**Authors:** Saba Vaezi, Moul Dey

**Affiliations:** School of Health and Human Sciences, South Dakota State University, Brookings, SD, USA

**Keywords:** Methadone maintenance treatment, Nutrition intervention, Dietary intake, Ultraprocessed, foods, Food-frequency questionnaire, Substance use disorder, Dietary, assessment, NOVA classification

Dear Editor,

We read with interest the randomized trial “*Nutritional intervention improved dietary intake among patients receiving methadone maintenance treatment*” (Ben Noach et al., 2026). Poor dietary quality and weight gain are common among individuals receiving methadone maintenance treatment (MMT) (Mahboub et al., 2021, Vallecillo et al., 2018), underscoring the need for approaches that extend beyond pharmacologic care. By examining nutrition within this setting, the study contributes useful evidence to an understudied area. Several methodological and interpretive points, however, would benefit from clarification to support interpretation and reproducibility.

The authors report reductions in total energy, carbohydrates, total fat, saturated fat, and ultra-processed food (UPF) intake in both arms. These estimates were derived from an Israeli Ministry of Health food-frequency questionnaire (FFQ) (Shahar et al., 2003), and additional detail on how frequency and portion-size responses were converted into quantitative nutrient values would help readers understand the analytic approach. Because this FFQ was originally developed and validated in older adults, additional information regarding its use and contextualization in this MMT population would aid interpretation.

The use of UPF as an outcome in a MMT population is relatively understudied and potentially valuable. However, the methods do not link the term to a specific classification system. NOVA is the most widely used framework, in which UPF corresponds to Group 4 and processed foods to Group 3 (Monteiro et al., 2019), yet the manuscript appears to use the two terms interchangeably. A brief description of the classification approach used in this study would aid interpretation and support future research.

The uncertainty about the dietary instrument also affects interpretation of the energy intake findings. The authors report reductions of roughly 1000 kcal/day in the intervention group and 1900 kcal/day in the control group, yet mean weight remained essentially unchanged in both arms over ten weeks. The reported reductions in energy intake appear large relative to the modest anthropometric changes observed during the intervention period and would benefit from additional context regarding dietary assessment and outcome interpretation. Information regarding intervention attendance and dietary adherence would also help contextualize the reported dietary changes.

We recognize that journal word limits constrain how much methodological detail can be included. Clarification regarding a few elements recommended by the CONSORT statement such as the prespecified primary outcome, sample-size determination, handling of missing data, and trial registration would further facilitate critical appraisal and reproducibility (Falci and Marques, 2015).

Overall, the study addresses an important and under-examined aspect of care for patients receiving MMT. By highlighting dietary quality and structured nutritional support, the authors draw attention to a dimension of recovery often overlooked in addiction care settings. Clarifying the methodological points above would further enhance the transparency and utility of these findings. We thank the authors for their contribution and look forward to continued work integrating nutritional care into addiction treatment settings.

## CRediT authorship contribution statement

**Saba Vaezi:** Investigation, Writing – original draft. **Moul Dey:** Investigation, Writing – review & editing.

## Author contributions

SV drafted the letter; all authors reviewed, critically edited, and approved the final version.

## Declaration of Generative AI and AI-assisted technologies in the writing process

During the preparation of this manuscript, the authors used ChatGPT (OpenAI) and Claude (Anthropic) to improve sentence structure and grammar. All content was subsequently reviewed and edited by the authors, who take full responsibility for the final version of the manuscript.

## Funding

This research did not receive any specific grant from funding agencies in the public, commercial, or non-profit sectors.

## Declaration of Competing Interest

The authors declare that they have no known competing financial interests or personal relationships that could have appeared to influence the work reported in this paper
